# Potential determinant factors of electronic cigarette regulation in Nigeria: qualitative interview of potential regulators

**DOI:** 10.1186/s12889-025-24376-7

**Published:** 2025-08-26

**Authors:** Anthony Weke, Colin Millard, Richard Holliday, Elaine McColl

**Affiliations:** 1 Department of Nursing, Midwifery and Health, Northumbria University, Coach Lane Campus, Benton, Newcastle-upon-Tyne, NE7 7XA UK; 2 Population Health Sciences Institute, Faculty of Medical Sciences, Newcastle University, Baddiley-Clark Building, Newcastle Upon Tyne, NE2 4AX UK; 3 School of Dental Sciences, Faculty of Medical Sciences, Newcastle University, Richardson Road, Newcastle upon Tyne, NE2 4AZ UK

**Keywords:** Electronic cigarettes, Regulations, Nigeria, Qualitative study

## Abstract

**Background:**

The World Health Organisation’s (WHO) 2021 report on the global tobacco epidemic highlighted dangers of e-cigarettes to youth and stated that countries without e-cigarette regulations leave themselves particularly vulnerable to the activities of tobacco and related industries. Nigeria, with a large proportion of young people, is now considering regulating e-cigarettes. This qualitative study aimed to provide insight into the factors that could influence such regulation.

**Methods:**

Three participants were recruited using purposive sampling and interviewed as representatives of potential Nigerian e-cigarette regulators. Interview transcripts were analysed using thematic analysis.

**Results:**

Five factors that could influence e-cigarette regulations in Nigeria were identified i.e., existing regulatory framework; research evidence; public health considerations; economic considerations; and e-cigarette industry.

**Conclusion:**

The young population of Nigeria and WHO recommendations on regulation of e-cigarettes provides stimulus for Nigerian authorities to impose some form of regulation of e-cigarettes in Nigeria. Adhering to the Nigerian regulatory framework requires compliance with existing policies, and multiple stakeholder consultation. The involvement of multiple stakeholders can be beneficial for inclusivity but may introduce three competing interests, public health, economic, and industry interest, which may conflict and unduly influence e-cigarette regulations in Nigeria. Regulators should prioritise regulatory measures that serve the most essential needs of the population. The findings of this research is beneficial to decision makers as it can help with regulatory preparedness by promoting factors with positive potential to help Nigeria meet its regulatory responsibilities while minimizing factors that do the opposite.

## Background

Reducing tobacco smoking is a public health priority globally, because tobacco use remains one of the world’s leading public health threats [1]. The advent of electronic cigarettes (e-cigarettes) has added a new dynamic to discussions about tobacco control measures. Although e-cigarettes do not contain tobacco, their role in affecting tobacco smoking has been the subject of many public health debates [2]. Some experts consider e-cigarettes to have the potential to compromise decades of public health efforts by acting as a gateway to traditional cigarettes and thereby driving smoking rates up, while others view them as an effective smoking cessation tool with the potential to drive smoking rates down [2]. This division amongst public health experts and researchers has led to considerable variation in regulatory approaches to e-cigarettes [3]. These variations can be placed on a spectrum ranging from health protection measures at one end, to harm reduction measures at the other. At the health protection end of the spectrum, policies are prohibitive (including complete bans on e-cigarettes) and aim to prevent new users from becoming addicted to nicotine. At the harm reduction end, policies are less restrictive and involve positive financial incentives to promote switching from tobacco cigarettes to a less harmful product (i.e., e-cigarettes) with the aim of reducing the more harmful toxicological effects of smoking tobacco cigarettes [4]. Internationally, e-cigarette regulations are driven by three main and contrasting principles: upholding consumer product standards; limiting access, addictiveness or attractiveness of e-cigarettes for youths and non-smokers; and facilitating e-cigarette use for smoking cessation by adults [5].

Amongst these three principles, it is likely that Nigerian e-cigarette regulations would be driven by the principle of limiting access, addictiveness or attractiveness of e-cigarettes for youths and non-smokers because Nigeria has a young population with approximately 43% under the age of 15 years [6]. It is likely to be vigilant in protecting a new generation of Nigerians against nicotine addiction and tobacco smoking with their associated health problems. According to WHO, nicotine is highly addictive and consumption in children and adolescents has negative impacts on brain development and can potentially lead to learning and anxiety disorders [7]. Young people can be initiated to nicotine uptake through experimentation with e-cigarettes [8]. Higher nicotine concentrations are associated with higher abuse potential and appeal of e-cigarettes [9]. The abuse potential may be the reason that 39 countries worldwide currently regulate the amount (concentration/volume) of nicotine in e-liquids [10]. However, regulators should balance nicotine restrictions with consideration that higher nicotine concentrations may help facilitate complete switching from cigarettes to e-cigarettes for adults using e-cigarettes as a smoking cessation aid [9].

Nigeria is a party to the WHO Framework Convention for Tobacco Control (FCTC) [11] and as such is likely to align itself with the guidance from WHO FCTC when regulating e-cigarettes. The WHO FCTC governing body, Conference of the Parties (COP), provides guidance on the regulation of tobacco and nicotine products [12]. COP activities and reporting around e-cigarette regulation to date are summarised in the WHO’s 2021 report [13] on the global tobacco epidemic which focused on new and emerging products, including e-cigarettes. It recommended that e-cigarettes should be strictly regulated for maximum protection of public health as they are addictive and because children and adolescents who use them can double their risk of smoking cigarettes [13].

Since Nigeria has now established regulatory and policy mechanisms to control the use and trade of tobacco products within the country (i.e. National Tobacco Control Regulations, 2019) [14], it may be time to also consider other novel products coming into the Nigerian market. A 2021 cross-sectional survey [15] showed an e-cigarette ever-use prevalence of 7.9% among the 949 respondents aged 15–35 years who were surveyed. As Nigeria looks to begin its e-cigarette regulatory journey, understanding the factors that could determine or influence the country’s e-cigarette regulations could help with regulatory preparedness by promoting factors with positive potential to help Nigeria meet its regulatory responsibilities while minimizing factors that do the opposite.

As this research project centred on e-cigarette regulation, three different theories of regulation were used to frame the findings on potential influencing factors of e-cigarette regulation in Nigeria. These theories are as follows. (1) Capture theory describes a situation whereby government regulatory agencies are gradually ‘captured’ by the regulated industry, so that, over time, they regulate primarily in the interest of industry, and not in public interest [16, 17]. (2) Expectation theory posits that the creation of expectations and promissory science around innovation among networks of medical professionals, research scientists and manufacturers influence the actions of regulatory agencies in favour of the innovation [16, 18–20]. (3) Corporate bias theory supposes that some organised interests can gain privileged access to executives and legislature, such that they are positioned to set the agenda for regulation, to bias it in favour of their interest at the expense of conflicting interest [16, 21].

## Methods

In this study, an interpretivist paradigm was adopted, and qualitative interviews were used to collect data from e-cigarette regulatory stakeholders in Nigeria. As the research was focused on the views of regulatory stakeholders, patients and the public were not involved. Purposive sampling was used to select three interviewees, who represented three organisations (Standards Organisation of Nigeria [SON], Federal Competition and Consumer Protection Commission [FCCPC], and Federal Ministry of Health, Nigeria [FMOH]). The selected organizations are strategic members of the Nigerian National Tobacco Control Committee (NATOCC), representing potential e-cigarette regulatory bodies. NATOCC is a committee established by Nigerian law under the National Tobacco Control Act, 2015, and is responsible for making recommendations for tobacco control policies to the Nigerian Minister of Health [22]. SON is the standardization body in Nigeria; it is legally mandated to prepare standards relating to products manufactured within or imported to Nigeria. FCCPC is the federal agency mandated to protect and promote the interest and welfare of consumers through initiation of policies relating to products and review of economic activities in Nigeria. FMOH is the government institution that develops and implements policies and programmes and undertakes other necessary actions to improve the health status of Nigerians.

Prior to commencement of this study ethical approval was granted by Newcastle University FMS REC (Date: 09/08/2022) and the National Health Research Ethical Committee of Nigeria (Date: 23/01/2023). As per interviewees’ preference, informed verbal consent was recorded prior to commencing the interviews. To gain access to rich and detailed information more rapidly and more effectively, a vignette [23] was used in this study; at the beginning of the interviews, the factors that were found to have influenced e-cigarette regulations in the US and the UK [24] were presented to interviewees to aid them in reflecting on factors that might come into play in the Nigerian context. All interviews were conducted online via Zoom by the lead author in February 2023. Inductive thematic analysis was carried out on interview transcripts using Braun and Clarke’s six-step process [25, 26].

## Results

Five themes were identified from the interviews: existing regulatory framework; research evidence; public health considerations; economic considerations; and e-cigarette industry.

### Existing regulatory framework

In this study, *existing regulatory framework* encompassed the current guidance and regulations (both internationally and Nigeria-specific) related to tobacco and related products that could be applied to or influence e-cigarette regulation. Three interviewees viewed EC as a product that should be classified as a tobacco product in Nigeria, and therefore subjected to the NTCR which currently regulates tobacco products in Nigeria:

*‘For us here*,* I think it* (e-cigarette) … *have to be regulated as tobacco product because*,* the position of the law*,* which is the legal provision in this sense now*,* is to the extent that it covers any product whether it is tobacco or tobacco related.’* (Representative of the Federal Competition and Consumer Protection Commission [RFCCPC], February 2023)

*‘The global direction is to regulate this product* (e-cigarettes) *as tobacco products. And when you regulate them as tobacco products*,* every aspect of tobacco regulation would come into play.’* (Representative of the Federal Ministry of Health, Nigeria [RFMOH], February 2023)

*‘Our standard calls them* (e-cigarettes) *tobacco and tobacco products… by the time you write tobacco and tobacco product*,* you now write what particular product you are referring to. It will now be an e-cigarette.’* (Representative of the Standards Organisation of Nigeria [RSON], February 2023)

RFMOH also identified the FCTC as another piece of existing guidance relevant to potential e-cigarette regulations:

*‘You know we are guided by the FCTC… And if the FCTC*,* which we are party to*,* is still cautious about these products* (e-cigarettes) … *the best way now is to go with the WHO FCTC.’* (RFMOH, February 2023).

### Research evidence

In this study, *research evidence* referred to the evidence base collected through research activities and data that would be used to inform decision making for policymakers and regulators of EC. RSON confirmed that their organisation would review available literature around EC and use the research evidence to shape their decisions:

*‘We must also dwell into other established literature… We look at the level of whether it’s* (e-cigarettes) *going to be harmful indeed*,* or it’s not going to be harmful*,* whether it’s going to be just as stated in the literature*,* established and validated literature*,* that it has a reduced harm compared to tobacco. So*,* all these things are part of things I’m saying*,* by the time we put them together*,* it will guide whatever position we need to take.’* (RSON, February 2023).

RSON further suggested that there are some aspects of e-cigarettes presented in the research literature that would need to be scientifically validated before they can be adopted into the Nigerian EC regulation:

*‘You can also take that sample*,* and then find a way to test it in the lab and confirm that those parameters or those requirements as stated there*,* when you get the—a result from the lab*,* you can compare those results with what the literature you have gotten.’* (RSON, February 2023).

### Public health considerations

In this study, *public health considerations* focused on the impact that EC use could have on the health of the population. Each of the interviewees viewed EC as a product that could potentially lead people - especially the vulnerable in Nigerian society (children and women) - to smoking:

*‘One of the things too that guides whatever we do*,* is the fact that we want to try as much as possible to discourage underage smoking. As well as smoking by the womenfolk… Underage smoking would be a very crucial one for us. If it looks like it’s something that would glamourise smoking—we would be very—quite wary of it.’* (RFCCPC, February 2023).

All three also expressed concerns that Nigeria lacks sufficient health services to tackle any possible public health challenge such as an epidemic of nicotine addiction or EC associated lung injuries that e-cigarettes may pose if they are not restricted in the country and prove to be more harmful than beneficial:

*‘So*,* that public health concern is one of the things that informs why we must regulate that product. More so that we also know that we have a situation where health services may not be as top notch. We are advancing bit by bit*,* but we don’t want a situation where something comes and it’s like an epidemic.’* (RFCCPC, February 2023).

The interviewees prioritized *public health considerations* as the most important factor that should influence regulatory decision making for EC:

*‘Globally*,* public health controls tobacco regulation because it’s an aspect that is topmost in terms of tobacco*,* public health… In any debate*,* with regards E-Cigarette in the world*,* the number one consideration is public health.’* (RFMOH, February 2023).

### Economic considerations

In this study, *economic considerations* related to the financial or commercial incentives for allowing the use of e-cigarettes in Nigeria and the benefit of setting out regulation for EC. The interviewees highlighted Nigeria’s obligations, both locally and internationally, to support commercial activities:

*‘Because we cannot impede trade as we are members of the WTO. Therefore*,* it becomes very important that we do not cause technical barrier to trade.’* (RSON, February 2023).

*‘So*,* for us*,* even outside of consumer rights*,* maybe right to choose and right to have access to a product that they desire to have*,* we also have an obligation to be responsible to the industry group.’* (RFCCPC, February 2023).

The interviewees also suggested that setting out regulation for EC in Nigeria would benefit the country by promoting industrialisation and boosting economic growth:

*‘The essence of the whole regulation is to give a clear level ground for both product that have been manufactured locally and those that are imported. Because if you don’t do that then you’ll be impeding trade*,* and therefore whoever is producing in the country it would be in the best interest of the country because it will add to the GDP of the country.’* (RSON, February 2023).

*‘If at all*,* those sciences (*that e-cigarettes are safer alternatives that are effective for smoking cessation*) are verified and then corroborated*,* we want a situation where we have additional*,* or rather we have advantages from people consuming them as there should also be something like production. So*,* we would be looking at tying*,* even licencing to production facilities that you have within the country… we are looking at how to promote industrialisation*,* how to create job opportunities for Nigerians and all of that.’* (RFCCPC, February 2023).

On the other hand, absence of EC regulation was viewed as economically detrimental to the country. For example, without guidelines, consumer demands for e-cigarettes can lead to illegal entry and illicit trade in e-cigarettes. Since such illicit trade does not follow the right channels, imported products are not taxed and revenues are not generated which, in turn, has economic implications. Poor border control in Nigeria increases the potential for illegal entry of e-cigarettes, and unregulated sales i.e., sales on the black-market:

*‘If we allow—because of our open borders*,* porous borders*,* these things* (e-cigarettes) *will continue to find their way into the country*,* and therefore as long as there is no regulations and no policy that will determine how these things—economically we are going to be losing.’* (RSON, February 2023).

The interviewees viewed these economic considerations as only secondary to public health considerations:

*‘The health component far outweighs any gain*,* any gain from any economic benefit that we could get… because all over*,* there have been several cases to that effect that have ruled*,* in favour of public health. That*,* public health supersedes any economic benefits a country can make.’* (RFMOH, February 2023).

### E-cigarette industry

In this study, *E-cigarette industry* encompassed how the activities of e-cigarette industry could potentially influence the e-cigarette regulatory process in Nigeria, including the direction of regulatory change. Vested interests of e-cigarette companies in investing in the Nigerian market might stimulate the development of e-cigarette regulations. They perceived that the availability of an e-cigarette regulatory framework would provide an appropriate legal framework and reassurance to enter and invest in the Nigerian e-cigarette market:

*‘Already we know that there are some companies who really wanted to start a business in Nigeria. And the only fear they have is that the issue of policy or regulation that*,* possibly have not been developed. But I’m—as I’m speaking*,* I have heard*,* that the responsible ministries have gone far*,* in ensuring that those policies are developed. Because no investors will want to come and invest in your country when there’s no policy framework and regulatory framework. This is one area that will scare all investors. So*,* for that reason*,* this I am very aware that something has started in that area.’* (RSON, February 2023).

Companies were also seen as likely to influence the process of regulatory change as it concerns e-cigarettes. From the interviewees’ perspective, the e-cigarette industry appears to be a major stakeholder in potential e-cigarette regulations in Nigeria.

*‘The industry can on their own*,* trigger an amendment. Like I say*,* even for instance if you—maybe the provisions that are there in the regulations for cigarette will not favour whatever they would do for the licencing of e-cigarette*,* it could raise some issues. And say okay look*,* we don’t think this is sufficient for you to use for the regulations of e-cigarette… this can’t happen like this and then certainly*,* we might have to have a consideration to say okay look*,* let us take these things back to the National Assembly.’* (RFCCPC, February 2023).

## Discussion of findings

### Existing regulatory framework

If, as seems likely from analysis of the interview transcripts, e-cigarettes were to be classified as a tobacco product in Nigeria, NTCR regulations [14] would apply to e-cigarettes; and by extension, guidance from the World Health Organisation (WHO) Framework Convention for Tobacco Control (FCTC) on e-cigarettes would also be taken into account. Nigeria is a party to the FCTC [11] and the NTCA [22] has as one of its main objectives to undertake activities recommended in the FCTC. Otherwise, if e-cigarettes were to be classified as non-tobacco products, a new regulation may be developed, the nature of which would depend on the actual specific classification of e-cigarettes, as seen in other countries e.g., classification as consumer products, devices, or combination products; medicines/drugs/medical devices; ENDS/e-cigarettes or vaping products; poisons or hazardous substances [10].

### Research evidence

The interviewees indicated that the outcome of reviewing the evidence and of testing it for validation processes would also inform the regulatory measures that would be applied to e-cigarettes in Nigeria. The interviewees appeared to be focused on whether e-cigarettes present substantial public health harms/risks (such as nicotine addiction, brain damage, increased smoking rate), or have a reduced harm/risk profile compared to tobacco cigarettes. In principle, the influence of research evidence could be related to an expectation theory [16, 18–20] i.e., networks of public health researchers and smokers facilitating a positive environment for the acceptance and approval of e-cigarettes because of expectations that e-cigarettes can help with smoking cessation. Despite this potential theoretical influence, in practice, a number of context–specific considerations are likely to oppose such influence. Firstly, in Nigeria, as of 2020, only 2.7% of people aged 15 years and older currently smoked (tobacco) cigarettes [27]. Given this low prevalence, interviewees suggested that Nigerian regulators will not prioritise the contribution of e-cigarettes to public health through their potential role as a smoking cessation aid. Moreover, e-cigarettes are a relatively expensive option for an average Nigerian. Conventional cigarettes, which are cheaper, still cost the average daily smoker in Nigeria just over 6% of their average annual income (measured by per capita GDP) [28].

Also, with regards to research in tobacco control in sub-Saharan Africa, the challenge of limited capacity to generate local research evidence exists [29]. The first and only national tobacco survey conducted to date in Nigeria is the Global Adult Tobacco Survey (GATS) collected in 2012 [30]. It remains to be seen whether Nigerian regulators will invest in generating research evidence or rely on evidence emanating from other countries to inform policy, considering that evidence from other countries may not always be applicable to the Nigerian context. With regards to independent testing of research evidence; implementation would be dependent on availability of funding for procurement of the equipment for independent testing. A 2018 study [31] on the analysis of tobacco control policies in Nigeria found that lack of funding slowed the policy implementation process [31]. It remains to be seen whether lack of appropriate infrastructure in Nigeria may have an influence on e-cigarette independent testing or regulatory process.

### Public health considerations

The interviewees suggested that there would be public health considerations of the potential impact of e-cigarette use in Nigeria. To the interviewees, America’s experience with e-cigarettes was marked by a crisis of nicotine addiction and perceived renormalisation of smoking. Interviewees suggested that, in Nigeria, public health considerations, and in particular concerns about e-cigarettes acting as a gateway product to tobacco, are likely to be the top priority when developing e-cigarette regulations because regulators would be cautious of avoiding regulatory actions that leads to increase in tobacco smoking. It is important to point out here that the gateway effect of e-cigarette has been criticized within the scientific community as lacking substantial evidence to establish causal relationship between vaping and smoking [32–34]. If the above public health consideration prevails, it places Nigeria at the health protection end of the regulatory spectrum i.e., e-cigarette regulation is likely to include prohibitive policies (potentially including complete bans on e-cigarettes) that aim to prevent new users from becoming addicted to nicotine [4]. However, historical experience has not shown public health considerations, including concerns about harms relating to addiction, to be the most influential factor affecting policy implementation in respect of other substances. For example, a study found that Nigeria’s limited alcohol-related policies and weak multi-sectoral action result from funding constraints and from the permissive culture for alcohol consumption in Nigeria posing a major limitation to enforcement; the alcohol Industry has leveraged this as a promotion strategy in Nigeria [35]. By contrast to its attitude toward alcohol consumption, Nigeria has an aversive culture for cigarette smoking, especially among young people [36]; this may provide an impetus for e-cigarette regulation.

In this study, the dominant public health consideration in Nigeria has to do with protection of non-smokers, especially children and women. The demographic profile of Nigeria’s population (in 2021, about 43.29% of Nigeria’s total population were children aged 0 to 14 years) [6] could be the reason why potential regulators there are so concerned with protection of children. In 56 countries worldwide, a minimum age restriction exists for the purchase and/or use of e-cigarettes [10]. In Nigeria, the NTCA [22] requires anyone selling or trading in tobacco products to verify the age of the purchaser (who should be at least 18 years old) by checking any form of official identification prescribed by law. The NTCA also prohibits sale or distribution of tobacco products through mainstream media, internet, or other online outlets where age verification can be challenging. These are some examples of regulatory measures that could be applied to e-cigarettes in Nigeria. The WHO Report on the Global Tobacco Epidemic, 2021 [13] shows that a ban on tobacco advertising, promotion, and sponsorship is enforced in Nigeria, albeit with an average compliance score of 4 out of 10 for all categories of legislated bans on tobacco advertising, promotion, and sponsorship.

### Economic considerations

Economic considerations were identified as likely to influence e-cigarettes regulations in Nigeria. Interviewees seemed to focus on obligations to different economic entities such as WTO and industry groups. Key economic considerations mentioned by interviewees were taxation and potential requirements for manufacturers to produce locally to benefit the economy. There are 35 countries worldwide that currently (July 2025) tax e-cigarettes; this is mostly through taxation of e-liquid and is based on its volume or price, but some countries tax the e-cigarette device and accessories as well. In 19 countries worldwide, specific excise tax based on volume is applied to e-liquids, while in 15 countries, an *ad valorem* (based on value/price) tax is used to tax e-cigarettes [10]. Economic measures could be used not only to produce economic gain but also to promote public health goals. For instance, governments argue that revenue generated from taxing tobacco products can be used to fund programs to buffer the health and economic impacts of smoking. This concept is referred to as ‘Hypothecation’ and means explicit earmarking of revenues from a particular tax so that they are only used for the advertised purpose [37]. A popular example is the ‘Sin Tax’ reform law initiated in the Philippines in 2012 which taxes alcohol and tobacco products with revenues earmarked and used to finance the Universal Health Care programme of the government [38].

### E-cigarette industry

In this study, it was identified that the e-cigarette industry may influence e-cigarette regulations in Nigeria. This may happen through regulatory capture, corporate bias or both. As discussed in the introductory section, regulatory capture may occur through e-cigarette industry influencing the Nigerian e-cigarette regulators, so that, over time, they regulate primarily in the interest of the e-cigarette industry, rather than in public interest [16, 17]. Whereas, with corporate bias, the e-cigarette industry may sponsor representation of their interest within the executive and legislative arms of government, such that the agenda for e-cigarette regulation is biased in the industry’s favour [16, 21]. Ideally, Nigeria should attempt to resist industry influence when e-cigarette regulations are made, but the experience with tobacco industry interference is not overly positive. In the most recent tobacco industry interference report, Nigeria’s rating dropped from 53 points in 2021 to 60 in 2023 mainly due to the government’s failure to adhere to transparency mechanisms mandated by the National Tobacco Control Act 2015 and the National Tobacco Control Regulations 2019 i.e., disclosure of demands made to industry or exchanges with the industry [39]. These breaches are reported to be exploited maximally by the industry to interfere in public health policy, and Nigeria is currently ranked 46 from a survey of 90 countries on tobacco industry interference [39].

### Limitations of this study

In this study, participants were members of the same committee (NATOCC) in the Ministry of Health and were officially shortlisted as representatives by the Ministry of Health to be interviewed for this study. This means that they worked closely with one another and so may be known to each other. Therefore, participants may know other interviewees. As a result, internal confidentiality, which involves the ability of participants in a research project to identify each other in the final publication of the research findings could not be guaranteed. However, external confidentiality was assured for all the participants who chose to be anonymized in the study. Also, presenting interviewees with a vignette that contains the factors that were found to have been taken into account in e-cigarette regulations in the US and the UK may have biased or limited the interviewees’ information on the potential influencing factors for Nigerian e-cigarette regulation. They may have looked at the questions primarily posed through the lens of events presented to have taken place in the US and or the UK. However, participants were directly asked about factors that may influence e-cigarette regulations in Nigeria but had not been found to have influenced regulations in the US and or the UK. Only three participants were interviewed in this study. However, these participants are representatives of the few organisations involved in regulation of tobacco and related products and were the only organisation put forward by the Nigerian Ministry of Health who acted as the gatekeeper to NATOCC in this study.

## Conclusion

Nigeria has a large population of young people who are vulnerable within society. The young population of Nigeria and WHO recommendations on regulation of e-cigarettes provides stimulus for Nigerian authorities to impose some form of regulation of e-cigarettes in Nigeria. Adhering to the Nigerian regulatory framework requires compliance with already existing policies, and multiple stakeholder consultation. The involvement of multiple stakeholders can be beneficial for inclusivity but may introduce three competing interests (i.e., public health, economic, and industry interest) which may conflict and unduly influence e-cigarette regulations in Nigeria. Notwithstanding, regulators should be prioritising regulatory measures that serve the most essential needs of the population. Evaluating available research evidence and assessing other country approaches against outcomes (such as, smoking cessation rates, rate of teenage use of e-cigarettes, smoking rates, etc.) provides a legitimate basis for regulatory choices.

## Data Availability

The datasets used and/or analysed during the current study are available from the corresponding author on reasonable request.

## Abbreviations

EC: Electronic cigarettes
WHO: World Health Organization
FCTC: Framework Convention for Tobacco Control
COP: Conference of the Parties
NTCR: National Tobacco Control Regulations, 2019
SON: Standards Organisation of Nigeria
FCCPC: Federal Competition and Consumer Protection Commission
FMOH: Federal Ministry of Health, Nigeria
RSON: Representative of Standards Organisation of Nigeria
RFCCPC: Representative of Federal Competition and Consumer Protection Commission
RFMOH: Representative of Federal Ministry of Health, Nigeria
NATOCC: Nigerian National Tobacco Control Committee
FMS: Faculty of Medical Sciences, Newcastle University
REC: Research Ethics Committee
NHREC: National Health Research Ethical Committee of Nigeria
US: United States of America
UK: United Kingdom
GATS: Global Adult Tobacco Survey
WTO: World Trade Organization
NTCA: National Tobacco Control Act, 2015
